# Comparing corneal biomechanic changes among solo cataract surgery, microhook ab interno trabeculotomy and iStent implantation

**DOI:** 10.1038/s41598-023-46709-5

**Published:** 2023-11-06

**Authors:** Shuichiro Aoki, Ryo Asaoka, Yuri Fujino, Shunsuke Nakakura, Hiroshi Murata, Yoshiaki Kiuchi

**Affiliations:** 1 Department of Ophthalmology, The University of Tokyo Graduate School of Medicine, Tokyo, Japan; 2 Department of Ophthalmology, Seirei Hamamatsu General Hospital, 2-12-12 Sumiyoshi, Naka-Ku, Hamamatsu City, Shizuoka Japan; 3 Seirei Christopher University, Hamamatsu City, Shizuoka Japan; 4 The Graduate School for the Creation of New Photonics Industries, Hamamatsu City, Shizuoka Japan; 5 Department of Ophthalmology, Shimane University Faculty of Medicine, Shimane, Japan; 6 Department of Ophthalmology, Tsukazaki Memorial Hospital, Hyogo, Japan; 7 Department of Ophthalmology, National Center for Global Health and Medicine, Tokyo, Japan; 8 Department of Ophthalmology and Visual Science, Hiroshima University, Hiroshima, Japan

**Keywords:** Ocular hypertension, Optic nerve diseases

## Abstract

Minimally invasive glaucoma surgery has expanded the surgical treatment options in glaucoma, particularly when combined with cataract surgery. It is clinically relevant to understand the associated postoperative changes in biomechanical properties because they are influential on the measurement of intraocular pressure (IOP) and play an important role in the pathogenesis of open-angle glaucoma (OAG). This retrospective case–control study included OAG patients who underwent cataract surgery combined with microhook ab interno trabeculotomy (µLOT group: 53 eyes of 36 patients) or iStent implantation (iStent group: 59 eyes of 37 patients) and 62 eyes of 42 solo cataract patients without glaucoma as a control group. Changes in ten biomechanical parameters measured with the Ocular Response Analyzer and Corneal Visualization Scheimpflug Technology (Corvis ST) at 3 and 6 months postoperatively relative to baseline were compared among the 3 groups. In all the groups, IOP significantly decreased postoperatively. In the µLOT and control groups, significant changes in Corvis ST-related parameters, including stiffness parameter A1 and stress‒strain index, indicated that the cornea became softer postoperatively. In contrast, these parameters were unchanged in the iStent group. Apart from IOP reduction, the results show variations in corneal biomechanical changes from minimally invasive glaucoma surgery combined with cataract surgery.

## Introduction

Glaucoma is one of the leading causes of blindness worldwide. Intraocular pressure (IOP) is the only modifiable established factor that halts the progression of glaucoma. The biomechanical properties of an eye are also important in the disease because they affect the measurement of IOP^1,2^, and moreover, they play an important role in the pathogenesis of glaucoma itself^3^. Advancements in biometric techniques in recent decades have enabled the direct measurement of biomechanical features of an eye. One of the most representative examples is the Ocular Response Analyzer (ORA; Reichert Inc., Depew, NY). ORA-measured corneal hysteresis (CH) reflects the damping capacity of the cornea^4–6^, which has been reported to be closely associated with the development^7^, severity^8^, and progression of glaucoma^9,10^. In addition, Corneal Visualization Scheimpflug Technology (Corvis ST; Oculus GmbH, Wetzlar, Germany) captures detailed images of the corneal deformation induced by the application of an air jet using an ultrahigh-speed Scheimpflug camera and provides data on various biomechanical parameters. Such Corvis ST parameters are associated with the severity^8^ and visual field progression^11^ of glaucoma.

Cataract surgery is often performed in aged patients. The prevalence of glaucoma also increases with increasing age^12,13^. Many studies have reported that IOP decreases after cataract surgery. However, a recent study indicated that this “IOP reduction” is not associated with the subsequent prevention of the progression of the disease^14^. We previously speculated that this is probably because of the biomechanical change in the eye following cataract surgery^15^. Currently, minimally invasive glaucoma surgery (MIGS) has greatly expanded the surgical treatment options in glaucoma, particularly when combined with cataract surgery. Thus, it is clinically relevant to understand the associated postoperative changes in biomechanical properties in relation to the change in IOP. However, there are few reports that shed light on this issue using ORA and Corvis ST.

The microhook ab interno trabeculotomy (µLOT) and iStent trabecular microbypass (Glaukos Corp., San Clemente, CA, USA) implantation procedures are two examples of MIGS, and recently, there has been an increase in the number of cases for both procedures^16,17^. Both techniques facilitate aqueous outflow, resulting in the reduction of IOP; the microhook incises the trabecular meshwork, whereas iStent is inserted into the trabecular meshwork. In the current study, the changes in IOP and the corneal biomechanical properties measured with ORA and Corvis ST were compared between µLOT combined with cataract surgery and iStent implantation combined with cataract surgery while matching background factors such as age and axial length; solo cataract surgery served as a control.

## Methods

This retrospective case‒control study was designed to investigate postoperative changes in biomechanical properties measured with ORA and Corvis ST in open-angle glaucoma (OAG) eyes after µLOT or iStent implantation combined with cataract surgery. Eyes receiving solo cataract surgery in patients without glaucoma were also included as controls. This study was approved by the Research Ethics Committees of the Seirei Hamamatsu General Hospital (#10619). This study was conducted in accordance with the tenets of the Declaration of Helsinki. All participants signed a written informed consent form for their clinical information to be stored in the hospital database and used for research.

### Participants

The study included OAG patients who underwent µLOT or iStent implantation combined with cataract surgery (µLOT and iStent group, respectively) and non-OAG eyes that received solo cataract surgery alone as a control group (solo cataract group). Age and axial length were matched among the 3 groups to avoid the possible effects of these variables on the biomechanical properties of the cornea. Inclusion criteria for the µLOT and iStent groups were as follows: (1) typical glaucomatous changes in the optic nerve head (e.g., rim notch with a rim width ≤ 0.1 disc diameter, a vertical cup-to-disc ratio > 0.7, or a retinal nerve fiber layer defect); (2) glaucomatous VF defects compatible with the optic nerve head changes meeting the Anderson–Patella criteria^18^ on two consecutive examinations; and (3) wide open angle with gonioscopy. The operation method chosen was based on the surgeon’s preference, but µLOT was selected when VF damage threatened the central VF, following the guidelines from the Japanese Ophthalmological Society^19^.

The solo cataract group included participants with no abnormal eye-related findings except for cataracts on biomicroscopy, gonioscopy, and funduscopy and no history of ocular diseases, such as diabetic retinopathy or age-related macular degeneration.

The exclusion criteria for the 3 groups were as follows: (1) contact lens wearers; (2) any abnormality of the cornea that could affect Corvis ST measurement, such as keratoconus; and (3) experience of any other ocular surgery, including corneal refractive surgery.

### Surgical technique

All procedures were performed by a single surgeon (RA) at Seirei Hamamatsu General Hospital between April 2020 and January 2022. All surgeries were performed under topical anesthesia. Phacoemulsification with intraocular lens implantation in the posterior chamber was performed through a clear corneal incision of 2.4 mm in length. After successful lens implantation involving eyes in the µLOT group, the trabecular meshwork was incised through two quadrants using a trabeculotomy microhook. In the case of eyes in the iStent group, the anterior chamber was filled with Opegan (Santen Pharmaceutical Co., Japan), and the iStent was injected under direct visualization into the nasal angle into Schlemm’s canal using a Swan-Jacob gonioprism lens (Ocular Instruments, Bellevue, WA, USA). Postoperatively, patients received both topical anti-inflammatory medication (Bromfenac Sodium Hydrate Ophthalmic Solution 0.1%, two times daily) and topical antibiotic (Moxifloxacin Ophthalmic Solution 1.5%, four times daily) for 6 weeks. All antiglaucoma medications prescribed preoperatively in glaucoma patients (in the µLOT or iStent group) were discontinued after the surgery. These medications were resumed at the discretion of the attending physician in the postoperative follow-up.

### Clinical data acquisition

Baseline demographic data regarding parameters such as age, sex, and axial length were collected from the medical charts. Axial length was measured preoperatively using the IOL Master ver. 5.02 (Carl Zeiss Meditec, CA, USA). Corvis ST, ORA, and Goldmann applanation tonometer (GAT)-IOP measurements were obtained preoperatively and at 3 and 6 months postoperatively. Corvis ST and ORA measurements were obtained at 15 min intervals. The order of the measurements was randomly determined.

### Corvis ST

**Figure 1:**
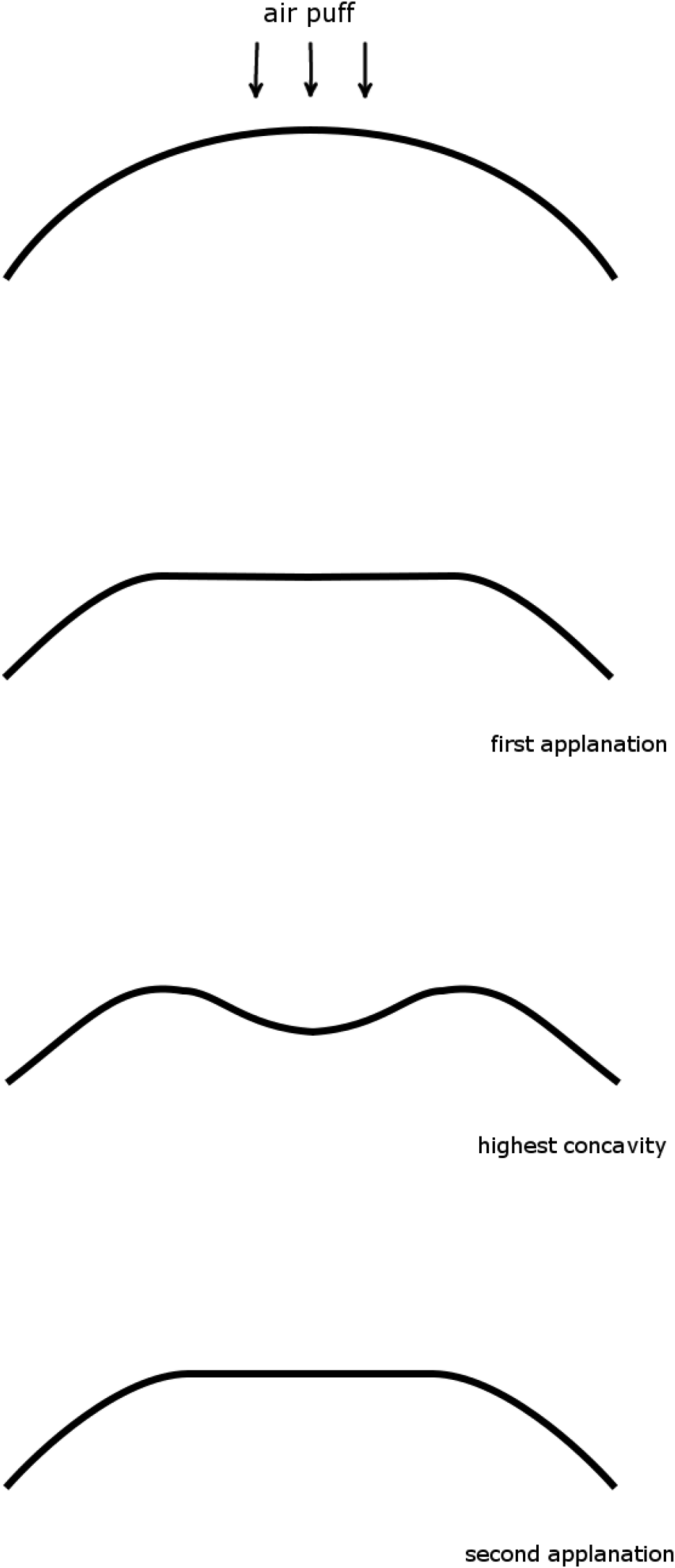
Corneal movement during Corvis ST measurement. From up to bottom: initial state before an air puff is applied; first applanation; highest concavity; second applanation.

**Figure 2:**
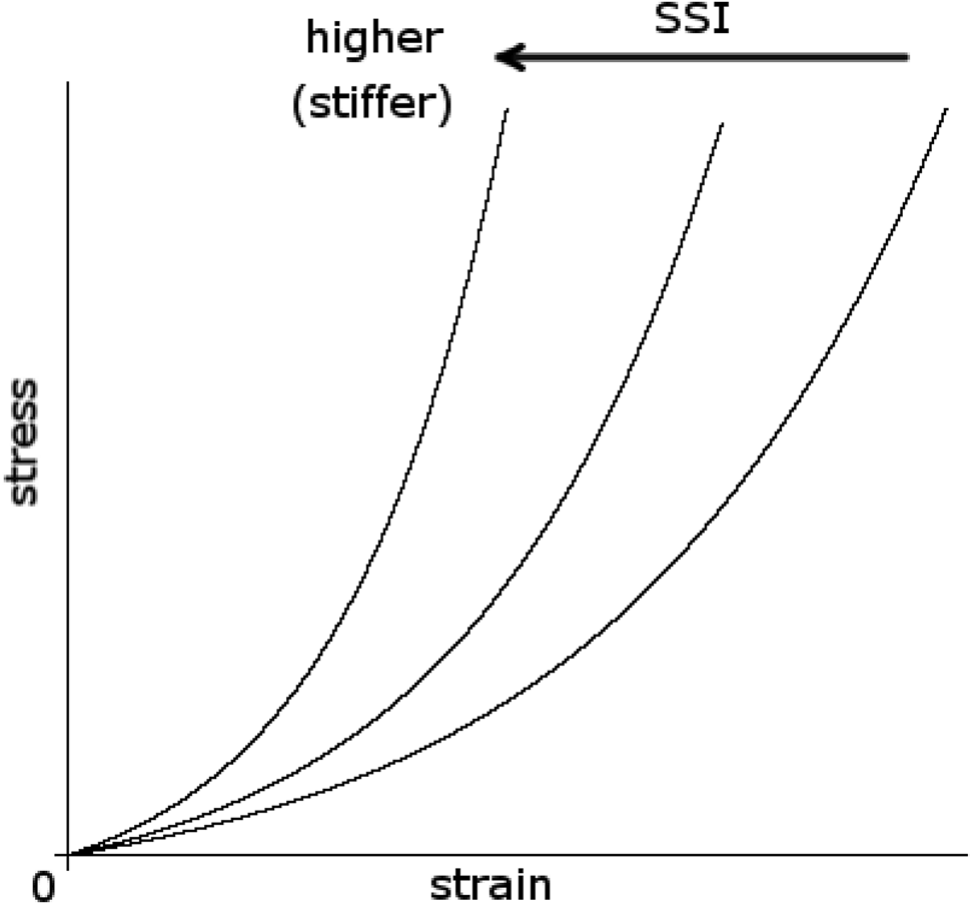
Stress–strain curves for different stress–strain index (SSI). A higher SSI value corresponds with a steeper curve.

The principles underlying Corvis ST measurements have been described in detail elsewhere^20^. In brief, a high-speed Scheimpflug camera records 140 images in 30 ms of corneal deformation in response to an air pulse emitted from the device. The response of the cornea is characterized by the moment when the cornea is most significantly depressed (highest concavity: HC) and by two moments of applanation, one during inward corneal movement (A1) and one during outward corneal movement (A2). Because the cornea is viscoelastic, it dissipates some of the applied energy, and the corneal shape at the second applanation during the outward motion of the cornea is different from that at the first applanation (Fig. 1). Based on recorded images, the Corvis ST device provides various raw parameters on morphology, such as the flatness of the deformed cornea, stiffness of the cornea, velocity of the corneal apex movement, and the timing of A1, A2, and HC (Table 1). All parameters were calculated using the current version of the Corvis ST software (version 1.6r2223). Corvis ST also provided more intuitive parameters calculated using the raw parameters. For instance, “biomechanical IOP (bIOP)” is the estimated IOP corrected for ocular biomechanical parameters^21^; “integrated inverse radius” is the integration of curvature during the concave state of the cornea. A higher integrated inverse radius indicates steeper indentation of the cornea, suggesting a softer cornea^22^. “Stiffness parameter A1” (SP-A1) and “stress‒strain index” (SSI) are also Corvis ST’s parameters related to the stiffness of the cornea. SP-A1 is the ratio of loading on the cornea to its displacement at the first applanation:

$$\mathrm{SP}-\mathrm{A}1=\frac{\mathrm{adjAP}1-\mathrm{ bIOP}}{\mathrm{A1DeflAmp}}$$

where adjAP1 represents the adjusted air pressure on the cornea at the first applanation and A1DeflAmp represents displacement of the corneal apex at the first applanation. A higher SP-A1 value indicates a stiffer cornea as more loading is needed to flatten the cornea^23^. As the formula suggests, SP-A1 represents a secant elastic modulus at a single state of the cornea, and the nonlinear relationship among IOP, corneal morphology, and the elastic properties of the cornea are not considered in the formula^4^. As a result, SP-A1 largely depends on IOP and corneal geometry (e.g., CCT). In contrast, the SSI is a measure of the nonlinearity of this stress‒strain relationship of the eye (Fig. 2). More specifically, SSI was developed using finite element methods so that it represents the “stiffness of cornea as material” independent from IOP and corneal geometry^24^. The SSI is shown on a normalized scale, where the average value of 50-year-old eyes is equal to 1. Higher SSI values indicate stiffer and less deformable corneas. Corvis ST measurements were conducted 3 times, and the average values were used for the analysis. Based on the quality indicator "OK" displayed on the instrument monitor, only reliable Corvis ST measurements were used.

**Table 1:** Summary of Corvis-ST measured parameters.

Name	Description
Biomechanical intraocular pressure (bIOP) (mmHg)	Corrected estimate of IOP obtained adjusted for ocular biomechanical parameters
A1 time (ms)	Time of the first applanation
A1 velocity (m/s)	Velocity of the corneal apex at the first applanation
A2 time (ms)	Time of the second applanation
A2 velocity (m/s)	Velocity of the corneal apex at the second applanation
Peak distance (mm)	Distance between nondeformed peaks
Deflection amplitude max (mm)	The maximum displacement of the corneal apex from baseline
Integrated inverse radius (mm^−1^)	The integration of curvature during the concave state of the cornea
Stiffness parameter A1 (SP-A1) (mmHg/mm)	The ratio of loading on the cornea over its displacement at the first applanation
Stress–Strain Index (SSI)	Represents material stiffness of the cornea

*IOP* intraocular pressure.

### ORA measurement

Details of the ORA measurement have already been summarized elsewhere^25^. In brief, an air pulse emitted from the instrument deflects the cornea, and the cornea experiences two applanations. After a certain time from the moment of the first corneal applanation, the jet flow decreases. Because the cornea is viscoelastic, some energy is dissipated, and the air jet pressure at the second applanation (P2), where the cornea is moving outward, is lower than the pressure at the first applanation (P1). The difference in pressure at these two applanations (P1-P2) is called CH. CH reflects the energy dissipation of the cornea^4–6^. The ORA measurement was measured in triplicate. Only measurements with a quality index of > 6.5 were used, and the average value was used in the analysis.

### Statistical analysis

Mean values of baseline clinical factors, Corvis ST, and ORA parameters were compared among the µLOT, iStent, and solo cataract groups with linear mixed models, where the patient was registered as a random effect because one or two eyes of a patient were included. The postoperative changes in GAT-IOP, Corvis ST and ORA parameters in each group were estimated using linear mixed models. P values were adjusted using the Benjamini‒Hochberg method for multiple comparisons.

All data processing and analyses were performed using the statistical programming language R (The R Foundation for Statistical Computing, Vienna, Austria).

## Results

Fifty-three eyes from 36 patients with OAG who had undergone µLOT surgery combined with cataract surgery, 59 eyes from 37 patients with OAG who had undergone iStent surgery combined with cataract surgery, and 62 eyes from 42 patients who had undergone solo cataract surgery were included in the study. In the µLOT group, 51 eyes of 34 patients had primary open-angle glaucoma (POAG), one eye had exfoliation glaucoma (EG), and one eye had steroid-induced glaucoma. In the iStent group, 56 eyes of 35 patients had POAG, and 3 eyes of 2 patients had EG.

Baseline characteristics as well as postoperative eye drop score and GAT-IOP of cases in each group are shown in Table 2. Age, axial length, CCT, and SP-A1 were similar among the 3 groups (all p > 0.05, linear mixed model with adjustment for multiple comparisons using the Benjamini‒Hochberg method). The baseline eye drop score was not significantly different between the µLOT and iStent groups (2.67 and 2.23 on average, respectively, p = 0.18). In contrast, the postoperative medication score was significantly higher in the µLOT group than in the iStent group at 3 (1.15 and 0.64 on average, respectively, p = 0.043) and 6 months (1.43 and 0.80 on average, respectively, p = 0.024). Baseline GAT-IOP was higher in the µLOT group than in the other two groups (p = 0.0018 and 0.023). On the other hand, GAT-IOP at 6 months postoperatively was similar among the 3 groups. Significantly lower CH, longer A1 and A2 time, smaller magnitude of A1 and A2 velocity, lower peak distance and deflection amplitude max were observed in the µLOT group than in the other two groups (all p < 0.05). In addition, the integrated inverse radius was significantly higher in the iStent group than in the solo cataract group (p = 0.016), and the SSI was significantly lower in the iStent group than in the other two groups (p = 0.047 and 0.0060, respectively).

**Table 2:** Basic characteristics and biomechanical parameters at baseline.

Parameter	μLOT group	iStent group	Solo-cataract group	p value (μLOT vs cat)	p value (iStent vs cat)	p value (μLOT vs iStent)
Age (years)	73.60 ± 9.05 [55.00 to 93.00]	75.81 ± 9.83 [51.00 to 91.00]	75.90 ± 8.21 [58.57 to 92.62]	0.30	0.96	0.34
Axial length (mm)	24.45 ± 2.24 [21.21 to 31.93]	24.44 ± 1.73 [21.12 to 28.62]	24.28 ± 2.24 [21.44 to 33.12]	0.78	0.78	0.96
Eye drop score (baseline)	2.67 ± 1.30 [1.00 to 5.00]	2.23 ± 1.25 [1.00 to 5.00]	–	–	–	0.18
Eye drop score (3 M)	1.15 ± 1.26 [0.00 to 4.00]	0.64 ± 0.98 [0.00 to 3.00]	–	–	–	0.043
Eye drop score (6 M)	1.43 ± 1.32 [0.00 to 5.00]	0.80 ± 1.24 [0.00 to 4.00]	–	–	–	0.024
GAT-IOP (baseline) (mmHg)	16.42 ± 3.73 [10.00 to 25.00]	14.66 ± 3.18 [9.00 to 23.00]	13.93 ± 3.19 [8.00 to 22.00]	0.0018	0.31	0.023
GAT-IOP (3 M) (mmHg)	12.55 ± 2.87 [8.00 to 20.00]	13.14 ± 2.96 [8.00 to 23.00]	11.72 ± 2.59 [7.00 to 19.00]	0.19	0.02	0.4
GAT-IOP (6 M) (mmHg)	13.30 ± 3.22 [8.00 to 20.00]	13.27 ± 2.78 [8.00 to 23.00]	12.40 ± 2.22 [8.00 to 18.00]	0.16	0.11	0.96
CH (mmHg)	8.92 ± 1.53 [4.61 to 13.28]	9.72 ± 1.63 [6.35 to 18.13]	9.93 ± 1.27 [6.94 to 12.69]	0.0033	0.58	0.025
CCT (μm)	524.92 ± 34.38 [456.00 to 598.00]	530.10 ± 37.21 [422.00 to 617.00]	536.34 ± 27.06 [471.00 to 590.00]	0.11	0.44	0.58
bIOP (mmHg)	13.64 ± 3.31 [8.70 to 25.00]	12.26 ± 2.53 [7.10 to 18.30]	12.38 ± 2.42 [6.20 to 17.70]	0.052	0.84	0.037
A1 time (ms)	7.27 ± 0.39 [6.50 to 8.70]	7.07 ± 0.33 [6.54 to 7.98]	7.05 ± 0.31 [6.34 to 7.70]	0.0071	0.85	0.016
A1 velocity (m/s)	0.15 ± 0.02 [0.09 to 0.19]	0.16 ± 0.02 [0.11 to 0.19]	0.16 ± 0.02 [0.11 to 0.18]	0.016	0.58	0.0069
A2 time (ms)	21.81 ± 0.61 [20.02 to 23.35]	22.20 ± 0.48 [21.37 to 23.35]	22.16 ± 0.46 [21.07 to 23.08]	0.0050	0.78	0.0033
A2 velocity (m/s)	− 0.27 ± 0.03 [− 0.34 to − 0.18]	− 0.28 ± 0.03 [− 0.36 to − 0.19]	− 0.29 ± 0.03 [− 0.35 to − 0.20]	0.0050	0.65	0.027
Peak distance (mm)	4.93 ± 0.32 [3.84 to 5.48]	5.15 ± 0.31 [4.09 to 5.75]	5.13 ± 0.29 [4.13 to 5.79]	0.0048	0.78	0.0033
Deflection amplitude max (mm)	0.96 ± 0.12 [0.61 to 1.21]	1.04 ± 0.12 [0.70 to 1.27]	1.01 ± 0.12 [0.76 to 1.28]	0.036	0.33	0.0033
Integrated inverse radius (mm^−1^)	8.70 ± 1.31 [5.95 to 11.34]	9.00 ± 1.00 [6.78 to 11.14]	8.49 ± 0.94 [6.79 to 10.98]	0.50	0.016	0.33
SP-A1 (mmHg/mm)	99.21 ± 20.53 [41.76 to 138.74]	93.18 ± 18.96 [61.08 to 141.51]	96.40 ± 19.47 [49.45 to 135.83]	0.58	0.50	0.22
SSI	1.28 ± 0.28 [0.90 to 1.86]	1.12 ± 0.20 [0.78 to 1.89]	1.21 ± 0.23 [0.77 to 1.79]	0.33	0.047	0.0060

p value: linear mixed model.

*bIOP* biomechanical intraocular pressure, *CH* corneal hysteresis, *CCT* central corneal thickness, *GAT* Goldmann applanation tonometry, *SP-A1* stiffness parameter applanation 1, *SSI* stress–strain index.

Postoperative changes at 3 and 6 months of GAT-IOP, Corvis ST, and ORA parameters in the µLOT group, iStent group, and solo cataract group are shown in Table 3 (A: µLOT group, B: iStent group and C: solo cataract group), whereas the comparisons of the postoperative changes at 6 months among the 3 groups are shown in Table 4. At 6 months postoperatively, eyes in both the μLOT and iStent groups exhibited significantly lower eye drop scores (− 1.00 and − 1.41, respectively, both p < 0.0001, linear mixed model with the adjustment for multiple comparisons using the Benjamini‒Hochberg method) compared to preoperatively, where there was no significant difference in the reduction of the scores between the two groups (p = 0.22, Table 4). GAT-IOP significantly decreased at 6 months postoperatively (− 3.11, − 1.39, and − 1.42 mmHg in the µLOT, iStent, and solo cataract groups, respectively, p < 0.0001, p = 0.015, and 0.00035, respectively) compared to preoperatively. The magnitude of GAT-IOP reduction at 6 months postoperatively was significantly greater in the µLOT group than in the iStent and solo cataract groups (p = 0.036 and 0.026, respectively, Table 4); however, there was no significant difference between the latter two groups (p = 0.96). bIOP also significantly decreased at 6 months postoperatively in all groups (− 2.12, − 1.15, and − 1.37 mmHg in the µLOT, iStent, and solo cataract groups, respectively, p < 0.0001, p = 0.0016, and p < 0.0001, respectively) compared to preoperatively, where there was no significant difference in the magnitudes of the bIOP reduction among the 3 groups (p = 0.18, 0.61, and 0.11, Table 4). CCT significantly increased at 6 months postoperatively in the µLOT group and iStent group (p = 0.0032 and 0.0016, respectively) but not in the solo cataract group (p = 0.77). CH showed a significant postoperative increase in the µLOT group (0.55 mmHg, p = 0.00026) but not in the iStent and solo cataract groups at 6 months postoperatively (− 0.19 and 0.21 mmHg, p = 0.54 and 0.19, respectively). There were different tendencies observed in the changes in Corvis ST parameters among the 3 groups; no significant changes in biomechanical parameters measured with Corvis ST were observed at 6 months postoperatively in the iStent group compared to preoperatively (Table 3). In contrast, in the µLOT and solo cataract groups, significant increases in A1 and A2 velocity, A2 time, peak distance, deflection amplitude max, and integrated inverse radius were observed at 6 months postoperatively compared to preoperatively; these changes indicated a more deformable cornea postoperatively. Consistent with this, significant decreases in SP-A1 and SSI were also observed in the µLOT (− 6.54 and − 0.20) and solo cataract (− 8.94 and − 0.06) groups at 3 and 6 months postoperatively (p = 0.019, p < 0.0001, p < 0.0001, and p = 0.018, respectively).

**Table 3:** Changes in biomechanical parameters 3 and 6 months after surgery in the (A) µLOT, (B) iStent, and (C) solo-cataract group.

Parameter	Δ(3M-preope)	p	Δ(6M-preope)	p	Δ(6M–3M)	p
(A) µLOT group						
Eye drop score	− 1.26 ± 1.45 [− 4.00 to 2.00]	< 0.0001	− 1.00 ± 1.38 [− 4.00 to 2.00]	< 0.0001	0.28 ± 0.93 [− 3.00 to 3.00]	0.04
GAT-IOP (mmHg)	− 3.87 ± 3.37 [− 15.00 to 4.00]	< 0.0001	− 3.11 ± 3.98 [− 15.00 to 6.00]	< 0.0001	0.63 ± 2.54 [− 6.00 to 6.00]	0.039
CH (mmHg)	0.39 ± 1.11 [− 2.83 to 4.98]	0.021	0.55 ± 0.96 [− 1.54 to 3.57]	0.00026	0.21 ± 0.87 [− 2.35 to 2.73]	0.2
CCT (μm)	3.36 ± 12.84 [− 25.00 to 48.00]	0.081	5.23 ± 11.44 [− 17.00 to 34.00]	0.0032	0.05 ± 10.83 [− 31.00 to 26.00]	0.22
bIOP (mmHg)	− 2.71 ± 2.77 [− 18.10 to 1.00]	< 0.0001	− 2.12 ± 2.93 [− 15.30 to 5.70]	< 0.0001	0.10 ± 1.41 [− 2.83 to 4.50]	0.039
A1 time (ms)	− 0.17 ± 0.36 [− 1.99 to 0.38]	0.0029	− 0.06 ± 0.41 [− 1.80 to 0.96]	0.31	0.06 ± 0.22 [− 0.33 to 0.68]	0.0044
A1 velocity (m/s)	0.02 ± 0.02 [− 0.01 to 0.11]	< 0.0001	0.02 ± 0.03 [− 0.05 to 0.08]	< 0.0001	− 0.001 ± 0.01 [− 0.03 to 0.03]	0.71
A2 time (ms)	0.41 ± 0.53 [− 0.69 to 2.62]	< 0.0001	0.29 ± 0.52 [− 0.77 to 2.09]	4.00E−04	− 0.01 ± 0.30 [− 1.06 to 0.52]	0.037
A2 velocity (m/s)	− 0.04 ± 0.04 [− 0.18 to 0.06]	< 0.0001	− 0.04 ± 0.04 [− 0.13 to 0.12]	< 0.0001	− 0.001 ± 0.03 [− 0.05 to 0.07]	0.79
Peak distance (mm)	0.29 ± 0.29 [− 0.18 to 1.62]	< 0.0001	0.26 ± 0.29 [− 0.33 to 1.27]	< 0.0001	− 0.01 ± 0.16 [− 0.36 to 0.34]	0.35
Deflection amplitude max (mm)	0.12 ± 0.11 [− 0.11 to 0.55]	< 0.0001	0.11 ± 0.12 [− 0.18 to 0.46]	< 0.0001	− 0.006 ± 0.07 [− 0.16 to 0.14]	0.71
Integrated inverse radius (mm^−1^)	0.44 ± 0.99 [− 1.54 to 2.88]	0.0037	0.67 ± 1.62 [− 2.55 to 8.65]	0.007	− 0.07 ± 0.78 [− 2.44 to 2.08]	0.28
SP-A1 (mmHg/mm)	− 11.78 ± 18.05 [− 77.94 to 26.39]	< 0.0001	− 6.54 ± 18.26 [− 57.65 to 36.52]	0.019	0.86 ± 7.83 [− 19.95 to 25.93]	0.011
SSI	− 0.18 ± 0.23 [− 0.89 to 0.30]	< 0.0001	− 0.20 ± 0.22 [− 0.87 to 0.27]	< 0.0001	0.02 ± 0.16 [− 0.35 to 0.43]	0.69
(B) iStent group						
Eye drop score	− 1.57 ± 1.31 [− 5.00 to 1.00]	< 0.0001	-1.41 ± 1.41 [− 5.00 to 2.00]	< 0.0001	0.15 ± 0.96 [− 2.00 to 4.00]	0.5
GAT-IOP (mmHg)	− 1.53 ± 3.08 [− 9.00 to 6.00]	0.0033	− 1.39 ± 3.29 [− 7.00 to 6.00]	0.015	0.63 ± 2.54 [− 6.00 to 6.00]	0.70
CH (mmHg)	− 0.11 ± 1.26 [− 8.40 to 1.57]	0.70	− 0.19 ± 1.26 [− 7.88 to 1.78]	0.54	0.21 ± 0.87 [− 2.35 to 2.73]	0.56
CCT (μm)	3.92 ± 21.13 [− 31.00 to 114.00]	0.43	7.33 ± 13.51 [− 18.00 to 51.00]	0.0016	0.05 ± 10.83 [− 31.00 to 26.00]	0.39
bIOP (mmHg)	− 1.11 ± 2.06 [− 7.30 to 3.30]	0.0016	− 1.15 ± 2.14 [− 7.60 to 2.50]	0.0016	0.10 ± 1.41 [− 2.83 to 4.50]	0.89
A1 time (ms)	− 0.04 ± 0.32 [− 0.75 to 0.55]	0.61	0.03 ± 0.33 [− 0.58 to 0.60]	0.69	0.06 ± 0.22 [− 0.33 to 0.68]	0.11
A1 velocity (m/s)	0.002 ± 0.02 [− 0.03 to 0.04]	0.66	0.003 ± 0.02 [− 0.05 to 0.05]	0.54	0.0009 ± 0.01 [− 0.03 to 0.03]	0.83
A2 time (ms)	0.05 ± 0.58 [− 1.61 to 1.04]	0.7	0.01 ± 0.55 [− 1.15 to 1.13]	0.95	− 0.01 ± 0.30 [− 1.06 to 0.52]	0.69
A2 velocity (m/s)	− 0.01 ± 0.04 [− 0.12 to 0.08]	0.43	− 0.01 ± 0.04 [− 0.11 to 0.08]	0.087	− 0.004 ± 0.03 [− 0.05 to 0.07]	0.66
Peak distance (mm)	0.03 ± 0.24 [− 0.50 to 0.71]	0.61	0.02 ± 0.23 [− 0.45 to 0.57]	0.67	− 0.01 ± 0.16 [− 0.36 to 0.34]	0.89
Deflection amplitude max (mm)	0.03 ± 0.11 [− 0.17 to 0.33]	0.18	0.02 ± 0.11 [− 0.23 to 0.26]	0.39	− 0.009 ± 0.07 [− 0.16 to 0.14]	0.66
Integrated inverse radius (mm^−1^)	0.17 ± 1.00 [− 2.28 to 1.94]	0.50	0.23 ± 1.10 [− 1.98 to 2.72]	0.39	− 0.07 ± 0.78 [− 2.44 to 2.08]	0.70
SP-A1 (mmHg/mm)	− 3.72 ± 18.12 [− 39.40 to 47.56]	0.39	0.15 ± 18.57 [− 45.13 to 38.18]	0.95	0.86 ± 7.83 [− 19.95 to 25.93]	0.087
SSI	− 0.02 ± 0.24 [− 1.02 to 0.52]	0.70	− 0.02 ± 0.22 [− 1.03 to 0.36]	0.69	0.02 ± 0.16 [− 0.35 to 0.43]	0.92
(C) Solo-cataract group						
GAT-IOP (mmHg)	− 2.12 ± 2.53 [− 7.00 to 4.00]	< 0.0001	− 1.42 ± 2.63 [− 7.00 to 5.00]	0.00035	0.63 ± 2.54 [− 6.00 to 6.00]	0.095
CH (mmHg)	0.003 ± 1.01 [− 2.24 to 2.87]	0.98	0.21 ± 1.04 [− 1.62 to 4.41]	0.19	0.21 ± 0.87 [− 2.35 to 2.73]	0.11
CCT (μm)	0.67 ± 16.14 [− 54.00 to 37.00]	0.81	0.73 ± 13.26 [− 51.00 to 25.00]	0.77	0.05 ± 10.83 [− 31.00 to 26.00]	0.98
bIOP (mmHg)	− 1.47 ± 1.89 [− 5.10 to 3.40]	< 0.0001	− 1.37 ± 1.84 [− 5.00 to 2.60]	< 0.0001	0.10 ± 1.41 [− 2.83 to 4.50]	0.74
A1 time (ms)	− 0.09 ± 0.27 [− 0.69 to 0.56]	0.026	− 0.03 ± 0.29 [− 0.75 to 0.59]	0.56	0.06 ± 0.22 [− 0.33 to 0.68]	0.087
A1 velocity (m/s)	0.01 ± 0.01 [− 0.04 to 0.04]	0.012	0.01 ± 0.02 [− 0.02 to 0.05]	0.003	0.001 ± 0.01 [− 0.03 to 0.03]	0.54
A2 time (ms)	0.18 ± 0.41 [− 0.82 to 0.95]	0.0026	0.17 ± 0.46 [− 1.06 to 1.10]	0.012	− 0.01 ± 0.30 [− 1.06 to 0.52]	0.8
A2 velocity (m/s)	− 0.01 ± 0.02 [− 0.07 to 0.03]	0.0016	− 0.01 ± 0.03 [− 0.11 to 0.04]	0.0037	− 0.0005 ± 0.03 [− 0.05 to 0.07]	0.94
Peak distance (mm)	0.09 ± 0.20 [− 0.31 to 0.50]	0.0017	0.08 ± 0.23 [− 0.45 to 0.67]	0.012	− 0.01 ± 0.16 [− 0.36 to 0.34]	0.77
Deflection amplitude max (mm)	0.05 ± 0.08 [− 0.13 to 0.23]	< 0.0001	0.05 ± 0.09 [− 0.15 to 0.29]	0.00071	− 0.004 ± 0.07 [− 0.16 to 0.14]	0.77
Integrated inverse radius (mm^−1^)	0.36 ± 0.85 [− 1.83 to 2.77]	0.0034	0.30 ± 0.79 [− 1.71 to 2.51]	0.011	− 0.07 ± 0.78 [− 2.44 to 2.08]	0.64
SP-A1 (mmHg/mm)	− 9.80 ± 14.06 [− 47.63 to 22.04]	< 0.0001	− 8.94 ± 14.92 [− 51.78 to 15.24]	< 0.0001	0.86 ± 7.83 [− 19.95 to 25.93]	0.55
SSI	− 0.08 ± 0.17 [− 0.49 to 0.30]	0.00097	− 0.06 ± 0.18 [− 0.50 to 0.42]	0.018	0.02 ± 0.16 [− 0.35 to 0.43]	0.35

p value: linear mixed model.

*bIOP* biomechanical intraocular pressure, *CH* corneal hysteresis, *CCT* central corneal thickness, *GAT* Goldmann applanation tonometry, *SP-A1* stiffness parameter applanation 1, *SSI* stress–strain index.

**Table 4:** Comparison of changes in different parameters at 6 months postoperatively among groups.

Parameter	Δ (6M-preope)			p value		
	µLOT group	iStent group	solo-cataract group	μLOT vs cat	iStent vs cat	μLOT vs iStent
Eye drop score	− 1.00 ± 1.38 [− 4.00 to 2.00]	− 1.41 ± 1.41 [− 5.00 to 2.00]	–	–	–	0.22
GAT-IOP (mmHg)	− 3.11 ± 3.98 [− 15.00 to 6.00]	− 1.39 ± 3.29 [− 7.00 to 6.00]	− 1.42 ± 2.63 [− 7.00 to 5.00]	0.026	0.96	0.036
CH (mmHg)	0.55 ± 0.96 [− 1.54 to 3.57]	− 0.19 ± 1.26 [− 7.88 to 1.78]	0.21 ± 1.04 [− 1.62 to 4.41]	0.13	0.11	0.003
CCT (μm)	5.23 ± 11.44 [− 17.00 to 34.00]	7.33 ± 13.51 [− 18.00 to 51.00]	0.73 ± 13.26 [− 51.00 to 25.00]	0.11	0.021	0.44
bIOP (mmHg)	− 2.12 ± 2.93 [− 15.30 to 5.70]	− 1.15 ± 2.14 [− 7.60 to 2.50]	− 1.37 ± 1.84 [− 5.00 to 2.60]	0.18	0.61	0.11
A1 Time (ms)	− 0.06 ± 0.41 [− 1.80 to 0.96]	0.03 ± 0.33 [− 0.58 to 0.60]	− 0.03 ± 0.29 [− 0.75 to 0.59]	0.69	0.35	0.26
A1 Velocity (m/s)	0.02 ± 0.03 [− 0.05 to 0.08]	0.003 ± 0.02 [− 0.05 to 0.05]	0.01 ± 0.02 [− 0.02 to 0.05]	0.0071	0.35	0.0029
A2 Time (ms)	0.29 ± 0.52 [− 0.77 to 2.09]	0.01 ± 0.55 [− 1.15 to 1.13]	0.17 ± 0.46 [− 1.06 to 1.10]	0.27	0.15	0.02
A2 Velocity (m/s)	− 0.04 ± 0.04 [− 0.13 to 0.12]	− 0.01 ± 0.04 [− 0.11 to 0.08]	− 0.01 ± 0.03 [− 0.11 to 0.04]	0.0013	0.95	0.003
Peak Distance (mm)	0.26 ± 0.29 [− 0.33 to 1.27]	0.02 ± 0.23 [− 0.45 to 0.57]	0.08 ± 0.23 [− 0.45 to 0.67]	0.0031	0.23	4.00E−04
Deflection amplitude max (mm)	0.11 ± 0.12 [− 0.18 to 0.46]	0.02 ± 0.11 [− 0.23 to 0.26]	0.05 ± 0.09 [− 0.15 to 0.29]	0.0065	0.25	0.00096
Integrated Inverse Radius (mm-1)	0.67 ± 1.62 [− 2.55 to 8.65]	0.23 ± 1.10 [− 1.98 to 2.72]	0.30 ± 0.79 [− 1.71 to 2.51]	0.21	0.74	0.17
SP-A1 (mmHg/mm)	− 6.54 ± 18.26 [− 57.65 to 36.52]	0.15 ± 18.57 [− 45.13 to 38.18]	− 8.94 ± 14.92 [− 51.78 to 15.24]	0.51	0.012	0.11
SSI	− 0.20 ± 0.22 [− 0.87 to 0.27]	− 0.02 ± 0.22 [− 1.03 to 0.36]	− 0.06 ± 0.18 [− 0.50 to 0.42]	0.003	0.35	0.00096

p value: linear mixed model.

*bIOP* biomechanical intraocular pressure, *CH* corneal hysteresis, *CCT* central corneal thickness, *GAT* Goldmann applanation tonometry, *SP-A1* stiffness parameter applanation 1, *SSI* stress–strain index.

All of the patients in the µLOT and iStent groups were prescribed topical prostaglandin analogs with or without other topical anti-glaucomatous drugs preoperatively. At 6 months postoperatively, 45 of 53 eyes (84.9%) in the µLOT group and 26 of 59 eyes (44.1%) in the iStent group were prescribed some kind of antiglaucoma topical medication. In particular, at 6 months postoperatively, 32 of 53 eyes (60.4%) in the µLOT group and 18 of 59 eyes (30.5%) in the iStent group were prescribed topical prostaglandin analogs.

## Discussion

In the current study, which included the matching of age and axial length among the three groups, postoperative changes in the corneal biomechanical properties measured with ORA and Corvis ST were examined in 112 OAG eyes that received cataract surgery combined with µLOT or iStent implantation and 62 eyes without OAG that received solo cataract surgery. As a result, a postoperative decrease in IOP was observed in all 3 groups. In addition, corneal stiffness (as suggested by the decrease in SP-A1 and SSI) decreased after µLOT and solo cataract surgery, whereas this was not the case in the iStent group.

Many studies have compared the biomechanical properties measured with Corvis ST between OAG eyes and normal eyes, providing different or partially contradictory results^26–32^. Many studies have found a longer A1/HC time, shorter A2 time, smaller magnitude of A1/A2 velocity, and smaller deformation amplitude (nearly equal to the deflection amplitude max plus posterior displacement of the whole eye) in POAG eyes than in normal eyes^26,27,31^. These reports indicated that corneas caved more slowly (longer A1/HC time and smaller A1 velocity) with smaller concavity (deflection amplitude max) and exhibited fast return to the initial state (shorter A2 time), which suggests stiffer corneas in OAG eyes than in normal controls. It should also be noted that all of these studies included treated OAG eyes. In contrast, it is also true that some case‒control studies have indicated softer corneas in OAG eyes. A softer cornea is represented by faster, greater and steeper indentation of the cornea, which corresponds to such parameters as smaller A1/HC/A2 time, greater peak distance, and larger integrated inverse radius and deflection amplitude ratio, respectively. Pillunat et al. found a smaller HC time and larger deflection amplitude ratio of the cornea at HC in treated normal tension glaucoma than in normal eyes^28^. Miki et al. reported a smaller A1/A2 time, larger peak distance, integrated inverse radius and deflection amplitude ratio in POAG eyes at the ‘treatment-naïve’ state than in normal eyes^29^. Another group also reported similar findings in treatment-naïve POAG^30^. These disagreements could partly be due to differences in the studied populations, such as disease subtype, IOP level, age, axial length, and the effect of antiglaucoma medications, all of which can affect Corvis ST measurements. Recently, Liu et al. conducted a meta-analysis of 15 case‒control studies, including the studies described above, reporting severe heterogeneities between the studies. They found lower A2 time and HC deformation amplitude suggestive of stiffer cornea in high-tension glaucoma (untreated IOP of more than 21 mmHg), whereas lower A1 time, HC time, and higher peak distance, suggestive of softer corneas, were observed in normal tension glaucoma than in normal controls^32^. In the current population at baseline, eyes in the µLOT group exhibited a significantly longer A1 time, shorter A2 time, smaller magnitude of A1 and A2 velocity, and deflection amplitude max than eyes in the solo cataract group, which are in agreement with the “stiffer cornea” findings in previous studies. This may be because the µLOT group included eyes with relatively high IOP (baseline GAT-IOP was 16.42 mmHg on average, Table 2); in a study on normal eyes, higher IOP was associated with the same direction of changes in A1/A2 time and the magnitude of A1/A2 velocity and deformation amplitude^33^. In contrast, a significantly higher integrated inverse radius and lower SSI were found in the iStent group than in the solo cataract group. The integrated inverse radius is the integral of the curvature of the cornea when it is concave, and the larger the value, the steeper the concavity of the cornea, suggesting a softer cornea. A lower SSI, a parameter of corneal stiffness independent of IOP^24^, also indicates softer corneas. These results are consistent with the “softer cornea” findings in normal tension glaucoma compared with normal controls that were previously reported^32^. Indeed, preoperative IOP was relatively low in the iStent group; this was similar to the solo cataract group while contrasting with the µLOT group.

Cataract surgery induces a reduction in IOP even in nonglaucomatous eyes (by 3.4 to 1.5 mmHg)^34–37^. In agreement with this, IOP was reduced by 1.42 and 1.37 mmHg for GAT-IOP and bIOP, respectively, in the solo cataract group (Table 3). The mechanism of the reduction in the IOP value following cataract surgery in nonglaucomatous eyes is yet to be fully understood; however, several mechanisms have been proposed. First, extraction of a thickened crystalline lens and implantation of a thinner intraocular lens changes the anatomic configuration of the anterior segment. Considerable lens volume reduction results in widening of the angle and facilitates aqueous outflow in eyes with relatively narrow angles^34^. It is also postulated that as the lens thickens, posterior traction on the scleral spur by the anterior lens zonules weakens and decreases outflow facility^37,38^. Cataract surgery may relieve this condition. Second, trabecular endothelium remodeling or metalloproteinase production may be induced due to stress from ultrasonic vibrations or high IOP during phacoemulsification, resulting in increased outflow facility^39,40^. In addition to these mechanisms for the “true” IOP reduction, other researchers using the Corvis ST measurements have proposed that the more deformable postoperative cornea was another reason^2,22,41^. In a prospective randomized trial^41^ that involved 93 normal eyes that underwent cataract surgery, it was found that bIOP and SP-A1 were decreased, whereas A1 and A2 velocity and deflection amplitude at HC were increased at 3 months postoperatively. In agreement with this, we observed significant increases in A1 and A2 velocity, peak distance, and deflection amplitude max postoperatively in the solo cataract group, suggesting a more deformable cornea in these eyes. Furthermore, two corneal stiffness-related parameters (SP-A1 and SSI) were decreased at 3 and 6 months postoperatively in the solo cataract group. SP-A1 is the ratio of the amount of stress over displacement of the cornea at the first applanation. Although SP-A1 is linearly corrected for bIOP (see the formula in “Methods”), it still depends on IOP because of the nonlinear material property of the cornea. In other words, the elastic modulus of the cornea depends on the external stress (i.e., IOP), which means that a higher IOP makes the cornea even less deformable than predicted by a simple linear stress‒strain relationship. On the other hand, SSI is a parameter of corneal stiffness that arises purely from material status and is independent of IOP^24^. The nonlinear stress‒strain property of an elastic material is represented as the steepness of the stress‒strain curve (Fig. 2), and the SSI is a parameter of the steepness. This parameter is calculated based on the results of the finite-element model experiment in which biomechanical responses of eyes were analyzed at different IOP levels. As a result, the reduction in SSI in the solo cataract group indicates a more deformable cornea independent of IOP. Thus, the decrease in both SP-A1 and SSI in the solo cataract surgery group suggested that the cornea becomes more deformable, even independent of the decrease in IOP. This is clinically very important when analyzing the effect of cataract surgery on glaucoma because the result is a possible underestimation of IOP after cataract surgery, as discussed above, and there is no settlement on whether postoperative IOP reduction is preventive against glaucoma progression. For instance, some previous reports have suggested that the rate of visual field progression does not change after cataract surgery in OAG eyes despite a decrease in IOP^42,43^. Furthermore, Kim et al. reported that visual field progression is faster after cataract surgery than preoperatively, which was attributed to postoperative IOP spikes^14^. These reports raise the question of whether the target IOP of the treatment should be altered after cataract surgery in OAG patients. Indeed, we previously reported that postoperative IOP reduction may not entirely be a true IOP reduction but may be the result of postoperative biomechanical changes, such as corneal softening^15^. The present study indicates that a similar finding can be observed in post-MIGS (µLOT) eyes; the cornea became more deformable in the µLOT group than in the solo cataract group as suggested by the higher reduction in SSI, deflection amplitude max, and peak distance (Table 4).

In the iStent group, there was a significant reduction in IOP following the surgery (− 1.39 mmHg at 6 months on average, Table 4), similar to the solo cataract group. Of note, SP-A1 and SSI were not significantly changed postoperatively in the iStent group, in contrast to the solo cataract group, which implies that the IOP reduction was not the result of the more deformable cornea. The magnitudes of IOP reduction were similar between the solo cataract and iStent groups (− 1.39 and − 1.42 mmHg, respectively, Table 4); however, this finding was associated with the reduction in medications in the iStent group (2.23 at baseline and 0.80 at 6 months postoperatively). These findings imply that the “reduction of IOP reading” was more due to “true IOP reduction” in the iStent group than in the solo cataract group. Whether this IOP reduction is associated with prevention of VF progression in glaucoma is a matter for further investigation in future studies.

Both iStent and µLOT are glaucoma surgeries targeted at facilitating aqueous outflow by decreasing the resistance of the trabecular meshwork; however, different findings were observed between their postoperative biomechanical properties. First, the magnitude of postoperative IOP reduction was significantly greater with µLOT than with iStent (− 3.11 and − 1.39 mmHg at 6 months postoperatively, Table 3B and C). However, one cannot simply draw a conclusion that µLOT is more effective than iStent in reducing IOP because preoperative IOP was also significantly different between the two groups (16.42 vs. 14.66 mmHg). Moreover, it should be noted that eyes in the μLOT group had a significantly higher postoperative eye drop score than those in the iStent group (1.43 vs. 0.80). Postoperative topical medication usage was sometimes resumed at the discretion of physicians to reach IOP targets. The relative contribution of antiglaucomatous medications to postoperative IOP reduction might be larger in the μLOT group than in the iStent group. In addition, unlike in the iStent group, both SP-A1 and SSI were significantly decreased postoperatively in the µLOT group, which suggests a more deformable cornea. The reason for this difference is not entirely clear; however, a few possible reasons may be applicable. First, the different changes in the Corvis ST parameters between the µLOT and iStent groups may be due to the different MIGS procedures. The resistance to aqueous humor outflow in the trabecular meshwork may be associated with the IOP rise during biomechanical measurements (induced by a sudden external force applied to the cornea). The trabecular meshwork has been reported to be stiffer and more resistant to external stress in eyes with glaucoma^44^. µLOT removes this resistance in a wider angle compared to iStent, and hence, an eye is likely to be less resistant to IOP increase when an external stress (air-puff) is applied in the former case. Additionally, removing a part of the trabecular meshwork, which may provide mechanical support for the limbal tissue, in the µLOT group could lead to a more deformable cornea postoperatively. The second possible reason is the effect of discontinuation of antiglaucoma medications. Antiglaucoma medications have significant effects on the biomechanical properties of ocular tissue, not only IOP. In particular, topical prostaglandin analogs induce corneal thinning^45–47^ and corneal softening^27,48^, probably through the activation of matrix metalloproteinase^49^. Yasukura et al. reported greater corneal compliance after prostaglandin analog use in glaucomatous eyes^50^. Wu et al. reported higher deflection amplitude measured with Corvis ST in POAG patients using topical prostaglandin analogs than in treatment-naïve POAG patients^27^. In the current study, all topical antiglaucoma medications, including prostaglandin analogs, were discontinued once postoperatively, and the corneal softening effect of the surgery may have been offset by the effect of discontinuing the use of topical prostaglandin analogs. At 6 months, most patients in the iStent group had not resumed usage of topical prostaglandin analogs at 6 months (18 eyes (30.5%) used topical prostaglandin analogs), whereas this was not the case in the µLOT group (32 eyes (60.4%) used topical prostaglandin analogs). This difference would have led to more pronounced corneal softening in the µLOT group than in the iStent group.

CH is a biomarker for the damping capacity of the cornea and is associated with the development^7^, severity^8^, and progression of glaucoma^9,10^. To our knowledge, changes in CH following the MIGS procedure combined with cataract surgery have yet to be described. In the current study, CH increased postoperatively at 6 months only in the µLOT group. In general, CH is negatively correlated with IOP^51^. Two groups found increased CH after trabeculectomy, where the magnitude of IOP reduction was 12.9 and 13.6 mmHg, respectively^52,53^. Another study reported increased CH after selective laser trabeculoplasty, which was discussed as a result of the reduced IOP (by 3.2 mmHg)^54^. However, another previous study^55^ reported unchanged CH 6 months after solo cataract surgery in glaucoma patients. In contrast, both in the iStent and solo cataract groups, no significant change in CH was observed at 3 and 6 months postoperatively. The change of CH in microLOT group in the current study can be considered as the result of both cataract surgery (corneal incision and replacement to IOL) and incision of trabecular meshwork. Previous studies indicated a larger postoperative change in CH after cataract surgery performed with a larger incision^56,57^, suggesting that surgical incision of the cornea affects corneal biomechanical properties. It is also possible that the small amounts of IOP reduction (less than 2 mmHg in GAT-IOP) in the iStent and solo cataract groups were not large enough to induce a detectable CH change in these groups. Another possible reason for this difference would be an effect of discontinuing antiglaucoma medications, particularly prostaglandin analogs. There are conflicting findings on the effect of topical prostaglandin analogs on CH. Tsikripis et al., in a randomized controlled trial, reported an increase in CH following the initiation of treatment with prostaglandin analogs in POAG patients^58^. In contrast, Meda et al., in a prospective case‒control study, reported a significant increase in CH after cessation of treatment with topical prostaglandin analogs^59^. In the current study, usage of antiglaucomatous medications was discontinued in many cases in the iStent and µLOT groups. Thus, the bias arising from the discontinuation of antiglaucoma medication usage on the postoperative change in CH could not be completely avoided in these groups.

CCT increased significantly at 6 months postoperatively in the µLOT and iStent groups (by 5.23 and 7.33 µm, respectively). This change is possibly due to corneal edema caused by surgically induced inflammation and the postoperative discontinuation of prostaglandin analogs^45–47^. Possibly, the latter effect may be more influential because the increase of CCT was not observed in the solo cataract group, and no considerable difference would be conceived in the postoperative corneal inflammation between the solo cataract surgery and iStent and µLOT surgeries. It has been reported that the GAT-IOP reading increased by approximately 0.32 mmHg per 10 μm increase in CCT^60^. In the current population, postoperative GAT-IOP reductions in the µLOT and iStent groups were − 3.11 and − 1.39 mmHg, respectively, which implies that all of them cannot be explained only by the increase in CCT (7.33 and 5.23 μm, respectively). The decrease in bIOP (IOP corrected for CCT^21^) also supports this speculation that the IOP reduction in both groups is independent of the change in CCT.

The present study has some limitations. The population was relatively small, and further validation of the current result in a larger dataset is needed. The second limitation arises from the nature of retrospective studies. Moreover, a future study should be conducted to assess the differences across different types of MIGS, such as 360-degree suture or Kahook dual-brade trabeculotomy^61^. µLOT and iStent implantation facilitate aqueous outflow by decreasing the resistance of the trabecular meshwork; however, it is not possible to separate the effect of µLOT and iStent implantation themselves on biomechanical properties from that of biomechanical property changes associated with cataract surgery because these MIGS techniques were performed in combination with cataract surgery; indeed, solo iStent surgery is not allowed in Japan^62^. Research should be conducted to compare the effect of solo-MIGS surgery in preparing such cases in the future. Additionally, the solo-cataract group, consisting of non-OAG patients without PGA usage, had different baseline characteristics compared to the other two groups, which consisted of OAG patients using PGA, which could affect corneal biomechanical properties. Comparing solo-cataract surgery with MIGS surgery in OAG patients might yield different results.

In conclusion, the current study suggested that corneas becomes more deformable after µLOT combined with cataract surgery in OAG eyes and cataract surgery in non-OAG eyes. These results indicate that "reduced IOP reading" after MIGS is influenced by changes in corneal biomechanics other than true IOP reduction and changes in the use of topical antiglaucoma medications. Such a finding was not observed with iStent combined with cataract surgery in OAG eyes.

## Data Availability

All data is available if requested to the corresponding author.
